# Pathogenic variants in the DEAH-box RNA helicase DHX37 are a frequent cause of 46,XY gonadal dysgenesis and 46,XY testicular regression syndrome

**DOI:** 10.1038/s41436-019-0606-y

**Published:** 2019-07-24

**Authors:** Ken McElreavey, Anne Jorgensen, Caroline Eozenou, Tiphanie Merel, Joelle Bignon-Topalovic, Daisylyn Senna Tan, Denis Houzelstein, Federica Buonocore, Nick Warr, Raissa G. G. Kay, Matthieu Peycelon, Jean-Pierre Siffroi, Inas Mazen, John C. Achermann, Yuliya Shcherbak, Juliane Leger, Agnes Sallai, Jean-Claude Carel, Laetitia Martinerie, Romain Le Ru, Gerard S. Conway, Brigitte Mignot, Lionel Van Maldergem, Rita Bertalan, Evgenia Globa, Raja Brauner, Ralf Jauch, Serge Nef, Andy Greenfield, Anu Bashamboo

**Affiliations:** 1 Human Developmental Genetics Unit, Institut Pasteur, Paris, France; 2 Department of Growth and Reproduction, Rigshospitalet, Copenhagen, Denmark; 3 School of Biomedical Sciences, Li Ka Shing Faculty of Medicine, The University of Hong Kong, Hong Kong, China; 4 Genetics and Genomic Medicine, UCL GOS Institute of Child Health, UCL, London, UK; 5 Mammalian Genetics Unit, Medical Research Council Harwell Institute, Oxfordshire, UK; 6 AP-HP, Hôpital d’Enfants Armand-Trousseau, Genetics and Embryology Department; Sorbonne Université; INSERM UMRS_933, Paris, France; 7 AP-HP, Hôpital Universitaire Robert-Debré, Pediatric Urology Department,; Reference Center for Rare Diseases (CRMR) Malformations Rares des Voies Urinaires (MARVU), Université de Paris, Paris, France; 8 Riley Children Hospital, Pediatric Urology Department; Indiana University, School of Medicine, Indianapolis, USA; 9 Genetics Department, National Research Center, Cairo, Egypt; 10 National Hospital, OHMATDYT, Kyiv, Ukraine; 11 Endocrinology et Diabetic Pediatrics, Hospital Robert Debre, Paris, France; 12 Second Department of Paediatrics, Semmelweis University, Budapest, Hungary; 13 Department of Pathology, University Hospital, University of Franche-Comté, Besançon, France; 14 Reproductive Medicine Unit, Institute for Women’s Health UCL, London, UK; 15 Department of Pediatrics, University Hospital, University of Franche-Comté, Besançon, France; 16 Human Genetics Center, University Hospital, University of Franche-Comté, Besançon, France; 17 First Department of Paediatrics, Semmelweis University, Budapest, Hungary; 18 Ukrainian Center of Endocrine Surgery Endocrine Organs and Tissue Transplantation, MoH of Ukraine, Kyiv, Ukraine; 19 Fondation Ophtalmologique Adolphe de Rothschild and Université Paris Descartes, Paris, France; 20 Department of Genetic Medicine and Development University of Geneva, Geneva, Switzerland

**Keywords:** disorders of sex development (DSD), testicular regression syndrome, DHX37, RNA helicase, ribosomopathy

## Abstract

**Purpose:**

XY individuals with disorders/differences of sex development (DSD) are characterized by reduced androgenization caused, in some children, by gonadal dysgenesis or testis regression during fetal development. The genetic etiology for most patients with 46,XY gonadal dysgenesis and for all patients with testicular regression syndrome (TRS) is unknown.

**Methods:**

We performed exome and/or Sanger sequencing in 145 individuals with 46,XY DSD of unknown etiology including gonadal dysgenesis and TRS.

**Results:**

Thirteen children carried heterozygous missense pathogenic variants involving the RNA helicase DHX37, which is essential for ribosome biogenesis. Enrichment of rare/novel DHX37 missense variants in 46,XY DSD is highly significant compared with controls (*P* value = 5.8 × 10^−10^). Five variants are de novo (*P* value = 1.5 × 10^−5^). Twelve variants are clustered in two highly conserved functional domains and were specifically associated with gonadal dysgenesis and TRS. Consistent with a role in early testis development, DHX37 is expressed specifically in somatic cells of the developing human and mouse testis.

**Conclusion:**

*DHX37* pathogenic variants are a new cause of an autosomal dominant form of 46,XY DSD, including gonadal dysgenesis and TRS, showing that these conditions are part of a clinical spectrum. This raises the possibility that some forms of DSD may be a ribosomopathy.

## INTRODUCTION

Disorders/differences of sex development (DSD) are defined as congenital conditions with discordant development of chromosomal and gonadal/anatomical sex and cover a wide range of phenotypes that involve the endocrine and reproductive systems.^1^ A subgroup of DSD consists of individuals with anomalies of testicular formation or the maintenance of testicular tissue during early embryonic development. The former comprises 46,XY complete or partial gonadal dysgenesis (MIM 400044). The complete form is characterized by female external genitalia, well-developed Müllerian structures, and gonads of fibrous ovarian-like stroma with no evidence of testicular differentiation.^2^ 46,XY partial gonadal dysgenesis is characterized by partially developed internal ducts usually consisting of a mixture of Wolffian (epididymis, vas deferens, and seminal vesicle) and Müllerian ducts (fallopian tube, uterus, and upper third of the vagina) with varying degrees of virilization of the external genitalia depending on the amount of testicular tissue present.^2^

46,XY testicular regression syndrome (TRS) is defined by a 46,XY chromosome complement, ambiguous or atypical genitalia, anomalies of sexual duct formation, and absence of gonadal tissue on one or both sides (MIM 273250).^3–5^ Some boys with TRS are born with normal external genitalia but present with cryptorchidism and may even have one or both palpable testes that subsequently involute. Children with TRS are considered to have variable degrees of testicular determination with the loss of gonad tissue early in gestation before testis formation is complete. The exact prevalence of TRS is unknown, but has been estimated to affect approximately 1:2000 boys.^6^ Anorchia is defined by the complete absence of testicular tissue in a 46,XY phenotypic male. However, since male-typical differentiation of the genital tract and the development of the external genitalia are dependent on the production of anti-Müllerian hormone (AMH) and androgens, the testis must have been present at least up to the 16th week of gestation in these individuals.^7^ In some circumstances anorchia may occur secondary to a vascular event or testicular torsion.^8^ Bilateral congenital anorchia has a prevalence of approximately 1:20,000 males and unilateral congenital anorchia a prevalence of 1:6500 males.^9^

Determining the genetic etiology of 46,XY gonadal dysgenesis and 46,XY TRS has been challenging. No gene pathogenic variants have been reported in association with the latter and in the former pathogenic variants in *SRY* (MIM 480000), *NR5A1* (encoding steroidogenic factor-1, SF-1; [MIM 184757]), and *MAP3K1* (MIM 600982) are the most prevalent causes, but together explain less than 40% of all 46,XY gonadal dysgenesis.^10^ Pathogenic variants in other genes including *SOX9* (MIM 608160), *SOX8* (MIM 605923), *GATA4* (MIM 600576), *DMRT1* (MIM 602424), *FOG2* (MIM 603693), *WT1* (MIM 607102), *DHH* (MIM 605423), *CBX2* (MIM 602770), *ATRX* (MIM 300032), *FGF9* (MIM 600921), and *ZNRF3* (MIM 612062) are reported in rare individuals as a cause of both syndromic and nonsyndromic forms of 46,XY gonadal dysgenesis as well as some 46,XY boys with severe penoscrotal hypospadias.^10–12^ The families of some patients with TRS raised as male also include other children with complete or partial 46,XY gonadal dysgenesis or agonadism who are raised as female.^4,5,13,14^ Thus, both 46,XY gonadal dysgenesis and TRS can be regarded as a continuum of phenotypes due to errors in testis determination and maintenance rather than clearly distinct and unrelated categories of atypical testicular formation.^14^ Though TRS is part of the clinical spectrum of 46,XY gonadal dysgenesis and may share a common genetic etiology, identification of the gene(s) involved has proven elusive.

Here, using exome and Sanger sequencing approaches for 145 individuals with 46,XY DSD of unknown etiology, we identified recurrent pathogenic variants in the DEAH-box RNA helicase *DHX37* specifically in association with 46,XY gonadal dysgenesis and 46,XY TRS.

## MATERIALS AND METHODS

### Patient and control samples

We studied 145 cases with 46,XY DSD of unknown etiology. They were defined as 46,XY gonadal dysgenesis (*n* = 81), 46,XY TRS (*n* = 16), 46,XY boys with severe penoscrotal hypospadias (*n* = 33), and 46,XY anorchia (*n* = 15). All patients were screened for pathogenic variants in genes known to cause DSD either by analysis of exome data sets or by direct Sanger sequencing of candidate genes. All patients with 46,XY DSD met the revised criteria of the Pediatric Endocrine Society (LWPES)/European Society for Paediatric Endocrinology (ESPE). This study was approved by the local French ethical committee (2014/18NICB—registration number IRB00003835) and consent to genetic testing was obtained from adult probands or from the parents when the patient was under 18 years. Patient ancestry was determined by self reporting, based on responses to a personal questionnaire, which asked questions pertaining to the birthplace, languages, and self-reported ethnicity of the participants, their parents, and grandparents. Genes known to be involved in 46,XY DSD were screened for pathogenic variants in the XY DSD cohort and high-resolution array comparative genomic hybridization (aCGH) was performed on all cases and indicated normal ploidy in all cases.

### Exome sequencing

One hundred patients presenting with 46,XY DSD were sequenced using the exome approach as described elsewhere.^11^ Exon enrichment was performed with Agilent SureSelect Human All Exon V4. Paired-end sequencing was performed on the Illumina HiSeq2000 platform with TruSeq v3 chemistry. Read files (fastq) were generated from the sequencing platform via the manufacturer’s proprietary software. Reads were mapped with the Burrows–Wheeler Aligner, and local realignment of the mapped reads around potential insertion/deletion (indel) sites was carried out with GATK version 1.6. Duplicate reads were marked with Picard version 1.62 (http://broadinstitute.github.io/picard/). Additional BAM file manipulations were performed with SAMtools (0.1.18). Single-nucleotide polymorphism (SNP) and indel variants were called with the Genome Analysis Toolkit (GATK) Unified Genotyper for each sample. SNP novelty was determined against dbSNP138. Novel variants were analyzed by a range of web-based bioinformatics tools with the EnsEMBL SNP Effect Predictor (http://www.ensembl.org/homosapiens/userdata/uploadvariations). All variants were screened manually against the Human Gene Mutation Database Professional Biobase (http://www.biobase-international.com/product/hgmd/). In silico analysis was performed to determine the potential pathogenicity of the variants. Potentially pathogenic variants were verified with classic Sanger sequencing.

### Sanger sequencing of the entire coding sequence of *DHX37*

Sanger sequencing was performed for the *DHX37* gene in 45 patients with 46,XY gonadal dysgenesis (*n* = 30), 3 patients with testicular regression syndrome, and 12 patients with anorchia. Polymerase chain reaction (PCR) amplification and Sanger sequencing of *DHX37* was performed using the primers listed in Table S1. The conditions for PCR were 95 °C for 5 minutes followed by 35 cycles of 95 °C for 30 seconds, 58.5 °C for 30 seconds, and 72 °C for 30 seconds. DNA sequence analysis was performed using at least 200 ng of purified DNA, 20 ng of primer and fluorescently labeled Taq DyeDeoxy terminator reaction mix (Applied Biosystems, Foster City, CA, USA) according to the manufacturer’s instructions. DNA sequence was determined using a 373A automated DNA sequencer (Applied Biosystems).

### Structural modeling

The homologous 2.6 Å crystal structure of Prp43-ADP-BeF3-U7-RNA complex (PDB: 5LTA)^15^ was used as a template to generate a homology model using SWISS-MODEL^15–20^ (https://swissmodel.expasy.org/). This model was chosen as the template because it had the highest score in models complexed with RNA and adenosine triphosphate (ATP) in the local alignment using BLAST (https://blast.ncbi.nlm.nih.gov/Blast.cgi) against the Protein Data Bank (PDB) database.^21^ Specific variants were made on the generated model using Chimera 1.11^22^ with the swapaa function. The single-stranded RNA motif and ADP-BeF3 of the 5LTA model was retained and used to view possible RNA and ATP contacts of the DHX37 pathogenic variants.

Annotations from UniProtKB (https://www.uniprot.org/uniprot/Q8IY37) and Pfam (http://pfam.xfam.org/protein/Q8IY37) were used as a guide to mark the domains in the structural model of DHX37. Some adjustments were made as compared with UniprotKB and Pfam based on the structurally defined domains of the DEAH-box family.^23^

### Collection of human fetal gonads and human immunohistochemistry

Human fetal gonads were isolated from material available following elective surgical termination of pregnancy during the first trimester at the Department of Gynecology at Copenhagen University Hospital (Rigshospitalet) and Hvidovre Hospital, Denmark. The regional ethics committee approved this study (permit number H-1-2012-007) and women gave their informed written and oral consent. None of the terminations were for reasons of fetal abnormality and all fetuses appeared morphologically normal. The fetuses in this collection were between gestational week (GW) 7 and 12, with fetal age determined by scanning crown–rump length. Fetal testis tissue was isolated in ice-cold phosphate buffered saline (PBS) and immediately fixed in formalin. Immunohistochemistry was conducted as previously described with anti-DHX37 (HPA047607, Sigma/Prestige Antibodies) diluted 1:50 and antigen retrieval in TEG buffer (10 mM Tris, 0.5 mM EGTA, pH 9.0).^24^ Immunofluorescence was conducted as previously described,^25^ with anti-DHX37 (HPA047607, Sigma/Prestige Antibodies) diluted 1:200 and anti-OCT4 (sc-5279, Santa Cruz Biotechnology) diluted 1:100. Immunofluorescence on the human cell lines RT4, KGN, and HEK 293 was performed as described elsewhere^10^ using anti-DHX37 (HPA047607, Sigma/Prestige Antibodies) diluted 1:200.

### Expression profiling in developing mouse gonads

For quantitative reverse-transcription PCR (qRT-PCR) of mouse tissue, messenger RNA (mRNA) was extracted from subdissected gonads of 6 XX and 6 XY 11.5 dpc mouse embryos using the RNeasy Plus Micro Kit (Qiagen). Each sample was then mixed with FastSYBR green master mix (Applied Biosystems) and either *Dhx37* or *Hrpt1* primers, plated in duplicate, and run on a 7500 Fast Real-Time PCR System (Applied Biosystems). Whole-mount in situ hybridization of 11.5 dpc wild-type (WT) XY gonads has been previously described.^26^ Immunofluorescence staining with anti-DHX37 (HPA047607, Sigma/Prestige Antibodies), anti-OCT4 (SC-5279, Santa Cruz Biotechnology), and DAPI (Vectashield with DAPI, Vector Laboratories) was performed on transverse sections of 11.5 dpc XY gonads. Sections were imaged at 20× using an LSM 700 Inverted Confocal Microscope (Zeiss).

### Site-directed mutagenesis

DHX37 expression vectors containing the all six missense pathogenic variants were generated by site-directed mutagenesis (QuikChange, Stratagene) with the use of WT DHX37 complementary DNA (cDNA) in both pAC-GFP and pCMV6-Myc expression vectors as templates. The entire coding sequence of all mutant plasmids was confirmed by direct sequencing prior to functional studies. The pcDNA-SOX9-Flag vector was a gift from Francis Poulat (Institut de Génétique Moléculaire de Montpellier, France).

### **S**ingle-cell array analysis of *Nr5A1*^+^, XY somatic cells of the developing murine gonad

We analyzed public single-cell RNA-seq (scRNA-seq) data from *Nr5A1*^+^ cells of the developing XY murine gonad as described elsewhere.^27^ The data correspond to expression of transcript with fkpm >1 at single-cell level in the developing male murine gonad at specific time points in embryonic development (E10.5, E11.5, E12,5, E13.5, and E16.5). The data were compiled into a spreadsheet that was used to generate a box and whisker plot using BioVinci data visualization package. The summary statistics used to create the box and whisker plot included the median of the data, the lower and upper quartiles (25% and 75%), and the minimum and maximum values array analysis.

### Cellular localization

Cellular localization of both WT-DHX37 and DHX37 mutant proteins were assayed by transfecting the different plasmids into HEK 293-T cells with the use of FuGENE HD (E231A, Promega) in chamber slides (C7182, 8 wells, Nunc) as described elsewhere^10^ using green fluorescent protein (GFP) signal from the DHX37-GFP WT and mutants, as well as antifibrillarin antibody (ab4566, Abcam) diluted 1:200. Images were obtained with a Leica Microsystems DMI4000B microscope at 100× magnification.

## RESULTS

### Genetic analysis of 46,XY DSD reveals *DHX37* pathogenic variants

**Fig. 1:**
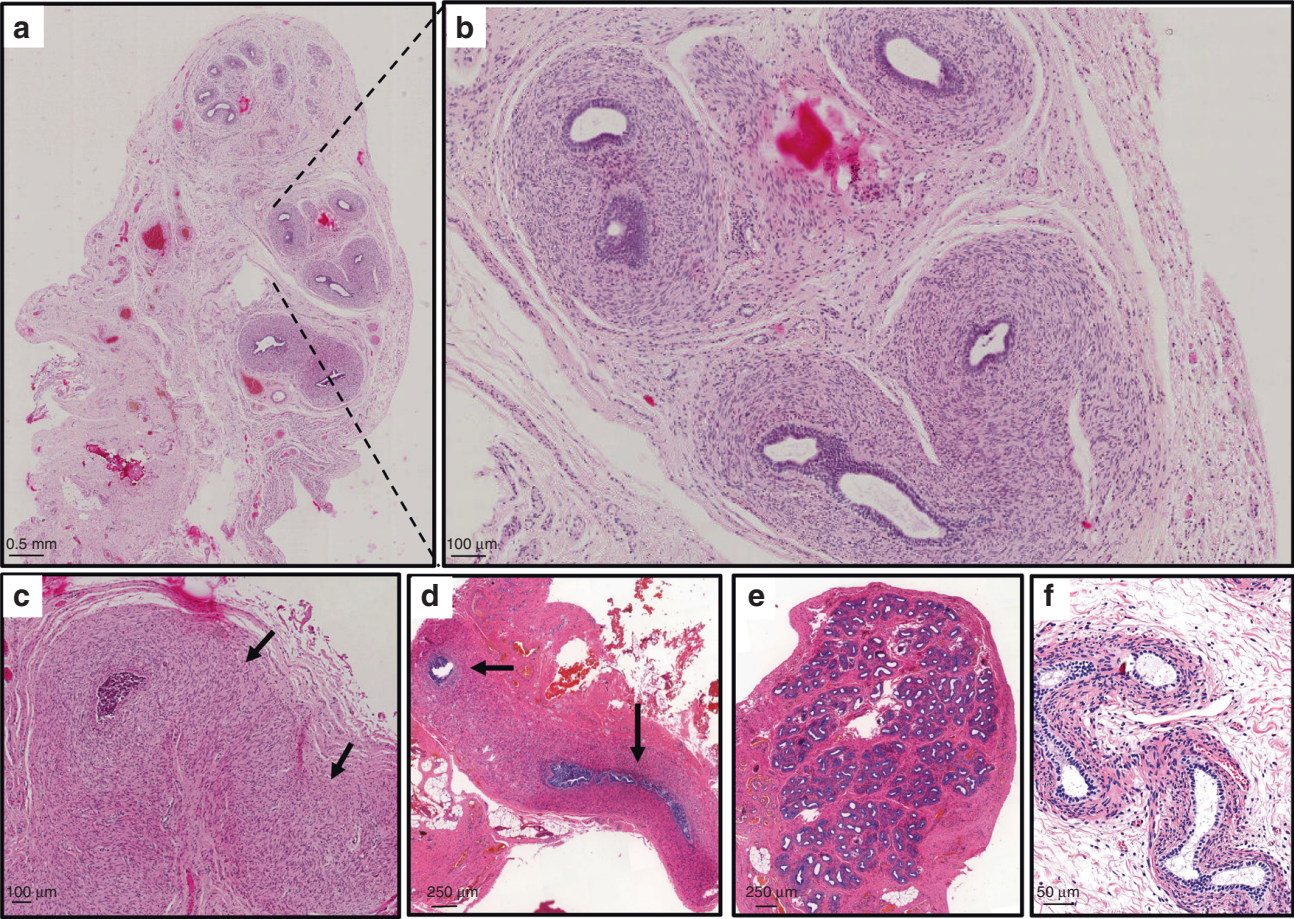
**Histologic analysis of gonad samples from three affected children.** (**a**) Right gonad of patient 12 with no evidence of testicular tissue and, at higher magnification in (**b**) epididymal-like structure. (**c**) Left “streak” gonad of homogeneous fibrous-like stromal tissue from the same child. (**d**–**e**) Histology from patient 10. No gonadal tissue was observed, however remnants of the ductus deferens were present on the sample from the right side (**d**, black arrows) and epididymal-like tissue was observed on the left side (**e**). (**f**) Epipidymal-like structures from patient 6. Size bars are indicated for each panel. Staining was performed with hematoxylin–eosin–saffron.

A genetic screen of 145 46,XY individuals with DSD of unexplained etiology identified 13 individuals who harbored recurrent and de novo heterozygous missense pathogenic variants involving six different amino acids in the putative RNA helicase DHX37. Pathogenic variants were specifically identified in children with 46,XY gonadal dysgenesis (9/81, 11%) and 46,XY TRS (4/16, 25%) but not in boys with either severe penoscrotal hypospadias or anorchia (Table 1 and 2). All amino acid changes, with the exception of the p.G1030E and p.R308Q pathogenic variants, are absent from public databases. The p.G1030E substitution is a very rare variant with a minor allelic frequency (MAF) of 4.9 × 10^−5^ in individuals of European ancestry (ExAC database http://exac.broadinstitute.org/), whereas the p.R308Q is absent from the ExAC database but is reported in one individual by genomic sequencing in the gnomAD database (http://gnomad.broadinstitute.org/). Combining both data sets indicates that this pathogenic variant has a MAF of 6.7 × 10^−6^. Five of six pathogenic variants analyzed were absent from either parent and are therefore de novo. One pathogenic variant, p.T304M was maternally inherited and the mode of inheritance in the remaining seven children is unknown because DNA from the parents was not available for study. The presenting phenotypes of children carrying pathogenic DHX37 pathogenic variants exist on a spectrum ranging from phenotypic females to males with bilateral or unilateral cryptorchidism (Table 1). However, of note the majority of pathogenic variants were associated with the presence of Wolffian and absence of Müllerian structures suggesting residual Sertoli and Leydig cell function during early testis formation (Fig. 1). No gonadal tissue was present following examination for four of the children. In all 13 children there was no evidence of any other somatic anomalies. All pathogenic variants were confirmed by Sanger sequencing.

**Table 1:** Phenotypes, genotypes, and investigation of gonadal function in 13 children with pathogenic variants in the *DHX37* gene

Variable	Patient 1	Patient 2	Patient 3	Patient 4	Patient 5	Patient 6	Patient 7	Patient 8	Patient 9	Patient 10	Patient 11	Patient 12	Patient 13
Sex of rearing	Female	Female	Female	Female	Female	Female	Female	Female	Female	Male	Male	Male	Male
Karyotype	XY	XY	XY	XY	XY	XY	XY	XY	XY	XY	XY	XY	XY
Age at presentation	20 years	3 years	16 years	15 years	15.5 years	Birth	NA	Birth	Birth	Birth	Birth	Birth	Birth
Diagnosis	Gonadal dysgenesis	Gonadal dysgenesis	Gonadal dysgenesis	Gonadal dysgenesis	Gonadal dysgenesis	Gonadal dysgenesis	46,XY DSD	46,XY DSD	Gonadal dysgenesis	TRS	TRS	TRS	TRS
Phenotype, external genitalia	Female, primary amenorrhea	Female	Female, primary amenorrhea	Female, discrete fusion of labia minora	Female, absence of puberty, discrete fusion of labia minora	Female, partial fusion of labia minora	Female, poorly developed labia	Virilized female, ambiguous genitalia	Ambiguous genitalia	Male, micropenis and bilateral cryptorchidism	Male, micropenis, hypospadias; unilateral cryptorchidism (L); small palpable testis (R)	Male, severe micropenis; cryptorchidism	Male, bilateral cryptorchidism
Internal genitalia	No Müllerian structures, Wolffian structures present	Urogenital sinus	No Müllerian structures, Wolffian structures present	Fallopian tube-like structures and epididymis on each side	Vagina present, absent uterus, Wolffian structures present (both sides)	Vagina 16–17 mm long and 6–7 mm wide not opened, absent uterus; no gonads present	Absent uterus and vagina	Vagina present, absent uterus, Wolffian structures present	Vaginal septum and uterus didelphys	No Müllerian structures	Vagina present	Absent vagina and uterus; bilateral epididymal-like structures	Absent gonads with vas deferens present (12 years)
Gonadal position	-	Abdominal cavity	-	-	-	-	-	-	-	L: Inguinal canal, R: abdominal cavity	-	-	-
Gonadal histology	Homogeneous fibrous tissues associated with a rete testis on both sides	Bilateral fibrous gonads, duct-like structures, fragments of uterine tube	R: Small nodule of fibrous tissue; L: fibrous tissue with rare tubule-like structures	No gonadal tissue present	Homogeneous fibrous tissue	L: No gonad tissue, remnants of ductus deferens; R: no gonad tissue, remnants of epididymis tissue	NA	NA	NA	No gonadal tissue; remnants of epididymis, ductus deferens	NA	L: Homogeneous fibrous tissue; R: no gonadal tissue	NA
**LH (U/liter)**													-
Value	23.1	7.2 (4 years)	18	17	20	0.7 (1 week) 3.3 (5 months)	NA	NA	NA	0.6 (1 week)	0.5 (28 days)	<0.4 (10 days)	NA
Reference range	2.4–13	Tanner 1: 0.03–0.55	2.4–13	2.4–13	2.4–13	0.2–0.5				0.2–0.5	0.2–0.5	0.2–0.5	
**FSH (U/liter)**													
Value	61.5	90.9 (4 years)	56	45	50	1.2 (1 week) 54.4 (5 months)	NA	NA	NA	10.7 (1 week)	8.9 (28 days)	26.7 (5 years)	100 (2 years)
Reference range	ND–13.5	0.7–3.39	ND–13.5	ND–13.5	ND–13.5	ND–2.0				ND–2.0	ND–2	ND–2	0.22–1.92
**T (ng/ml)**													
Value	0.29	<0.025 (4 years)	<0.25	<0.25	<0.25	0.25 (1 weeks) <0.19 (5 months)	NA	NA	NA	0.16 (1 weeks) 2 (9 weeks)	0.1 (Basal and after HCG 1500 ui x3)	0.2 (10 days)	0.7 (11 years)
Reference range	2.70–9.00	0.02–0.23	2.70–9.00	2.70–9.00	2.70–9.00	0.1–0.5 0.02–0.2				0.1–0.5 0–8–3.3	0.2–0.5	0.7–1.7	(0.4–19)
**AMH (ng/ml)**													
Value	0.29	0.06 (4 years)	NA	0.65	NA	0.01 (5 months)	NA	NA	NA	NA	NA	0.0	<0.01 (11 years)
Reference range		32.77–262.69				105–270							7.4–243
Ancestry	European	European	European	European	European	European	European	European	European	European	European	European	European
DHX37 pathogenic variant and inheritance	c.G2021A p.R674Q unknown	c.G2021A p.R674Q de novo	c.G923A p.R308Q de novo	c.C911T p.T304M maternal	c.C911T p.T304M de novo, absent in unaffected XY sib	c.G1001T p.R334L de novo	c.G923A p.R308Q unknown	c.G923A p.R308Q unknown	c.G923A p.R308Q unknown	c.C1877T p.S626L unknown	c.C1000T p.R334W unknown	c.G923A p.R308Q de novo	c.G3089A p.G1030E unknown

Reference range refers to the range of basal levels in control subjects matched according to age and chromosomal sex with the case subjects. To convert the values for testosterone to nanomoles per liter, multiply by 3.467. To convert the values for estradiol to picomoles per liter, multiply by 3.671.

*AMH* anti-Müllerian hormone, *DSD* disorders/differences of sex development, *FSH* follicle stimulating hormone, *L* left, *LH* luteinizing hormone, *NA* not available, *R* right, *T* testosterone, *TRS* testicular regression syndrome.

**Table 2:** Summary of the frequency of *DHX37* pathogenic variants found in association with each 46,XY DSD subtype

Phenotype	DHX37 Amino acid change	Frequency
46,XY Gonadal dysgenesis *(n* = 81)	p.T304M (2), p.R308Q (4), p.R334L, R674Q (2)	9/81 (11%)
46,XY Testicular regression syndrome (*n* = 16)	p.R308Q, p.R334W, p.S626L, p.G1030E	4/16 (25%)
46,XY DSD (boy with penoscrotal hypospadias) (*n* = 33)	-	0
46,XY Anorchia (*n* = 15)	-	0

*DSD* disorders/differences of sex development.

*DHX37* is one the most highly conserved genes in the human genome and is intolerant to loss-of-function and missense variants in the general population.^28^ Fisher’s exact test (two-tailed) on the frequency of loss-of-function and missense variants observed in our 46,XY DSD cohort, compared with rare (MAF < 0.01%) missense variants in the *DHX37* gene from 32,500 control individuals of matched-ancestry (ExAC database), shows a highly significant enrichment of rare missense variant in the *DHX37* gene in the overall DSD cohort (*P* value = 5.8 × 10^−10^). The association with the subgroups of 46,XY gonadal dysgenesis and 46,XY TRS is even more striking (*P* value = 6 × 10^−12^). If we consider the five de novo missense pathogenic variants in *DHX37* and compare this with the frequency of de novo missense *DHX37* variants in 2278 control individuals (denovo-db.gs.washington.edu), the DSD cohort is highly enriched for de novo variants (*P* value = 1.5 × 10^−5^). This may indeed be an underestimation because the inheritance of the *DHX37* pathogenic variant was unknown in 7/13 children. Furthermore, in the family of patient 5, the pathogenic variant was both de novo and absent from the unaffected 46,XY brother.

**Fig. 2:**
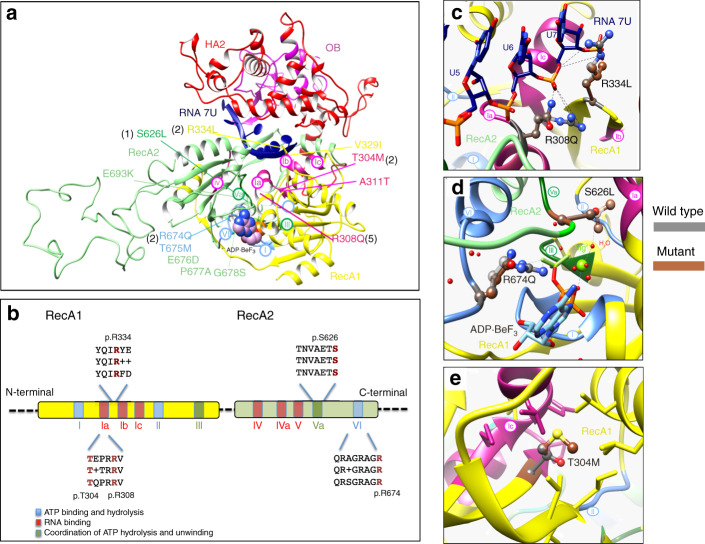
**In silico modeling of DHX37 RecA1 and RecA2 pathogenic variants.** (**a**) Functional domains of a homology model of DHX37 protein with U_7_-RNA (dark blue) and a nonhydrolyzable adenosine triphosphate (ATP) analog (ADP-BeF_3,_ spheres). Domains are color-coded and labeled, conserved motifs are specified in circles, and the five disease-associated variants are indicated. The protein has four functional domains: two RecA-like domains, which are the helicase domains (RecA1: ATP-binding DEAH-box helicase, yellow, RecA2: C-terminal helicase, green); helicase associated 2 domain (HA2, red); and oligonucleotide/oligosaccharide-binding like domain (OB, pink). (**b**) Schematic diagram of the RecA-like domains in DEAH-box RNA helicases. Colors represent main helicase functions. Alignment of human (top) and *Saccharomyces cerevisiae* (bottom) showing the positions of recurrent variants causing 46,XY disorders/differences of sex development (DSD) with the consensus sequence shown in the middle. (**c**) Zoomed-in view of residue 308 and 334 with single-stranded RNA. (**d**) Residues 626 and 674 are shown to interact with the ATP analog. (**e**) Residue 304 is highlighted to be buried with a pocket within the RecA1 domain. Dashed lines within figure parts are shown for selected noncovalent polar interactions.

Interestingly, with one exception, all pathogenic variants are clustered within the functional RecA1 and RecA2 domains of the protein and involve amino acid residues that are highly conserved through to yeast (Fig. 2a and Supplemental Fig. 1). Consistent with this finding, all amino acid changes are considered to be highly damaging by multiple predictive software programs. Eleven of the 13 children have pathogenic variants specifically in four amino acid residues. Five children were mutated for the R308 residue and two children each for the T304, R334, and R674 residues respectively. None of the 13 children carrying pathogenic variantss in *DHX37* carried pathogenic variants in other genes known to cause 46,XY DSD.

### In silico conservation and modeling of DHX37 amino acid substitutions

RNA helicases are characterized by a highly conserved core domain of two tandem RecA-like domains (RecA1: ATP-binding DEAH-box helicase, RecA2: C-terminal helicase), which are connected by a flexible linker (Fig. 2a).^29,30^ Within these domains are 11 sequence motifs conserved throughout to yeast, which are involved in RNA substrate interaction, nucleoside triphosphate (NTP) binding and hydrolysis, and the coordination of unwinding activity. Four of the pathogenic variants fall within the motifs Ia, Va, and VI, which have been implicated in RNA binding, coordination of ATP binding and unwinding, and ATP binding and hydrolysis respectively (Fig. 2b). To predict the molecular consequences of the pathogenic variants we generated homology models of DHX37 using a crystal structure of the DEAH-box helicase Prp43 (PDB ID:5LTA) in an active state complexed with single-stranded U_7_-RNA and a nonhydrolyzable ATP analog (ATP-BeF_3_).^15^

The pathogenic variants p.R308Q, p.R334L are located at the DNA/RNA contact interface and provide an electrostatic environment to accommodate nucleic acids (Fig. 2a, c). These two arginines (R308 and R334) are highly conserved within the DEAH-box family and their counterparts in Prp43 interact with the RNA backbone.^15^ The recurrent p.R308Q pathogenic variant found within the conserved sugar-phosphate binding site, Ia, removes a positive charge and likely weakens this interaction.^31^ The p.R334L substitutions a hydrophobic leucine at conserved positions otherwise occupied by charged or polar amino acids with predicted detrimental effects on DNA/RNA-binding. The variants p.S626L and p.R674Q map to sequence motif Va or VI, respectively, which are both involved in ATP binding, hydrolysis, and cross-talk with the unwinding machinery (Fig. 2d). Serine 626 interacts with water and magnesium in the active site and arginine 674 forms electrostatic interactions with the γ-phosphate of ATP. The residue p.T304 of motif Ia is not directly involved in RNA binding but mediates intramolecular packing, which would be affected by the bulkier methionine side-chain introduced in the p.T304M pathogenic variant (Fig. 2e). Further evidence in favor of causality of these pathogenic variants is indicated by the analysis of seven missense variants reported in the gnomAD/ExAC databases that are present in low frequencies in the general population and may not be pathogenic (Table S2). These are located in the RecA1 and RecA2 domains. In silico modeling of all seven low frequency non-pathogenic variants indicated that in contrast to the pathogenic variants, they are predicted not to have either major structural or functional consequences (Supplemental data and Fig S2).

### Expression analysis of DHX37 in early gonad development and functional studies

**Fig. 3:**
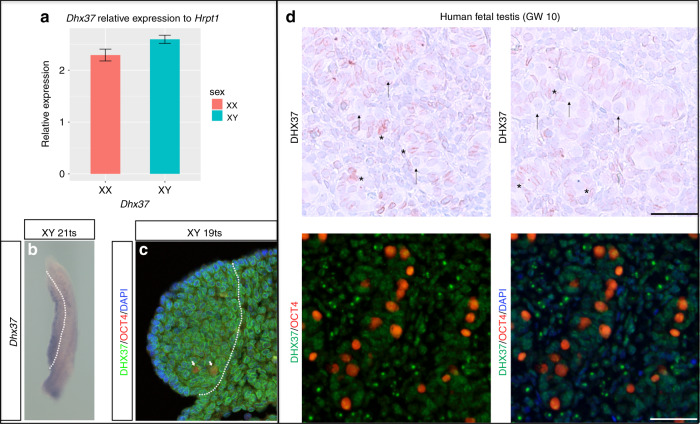
**Expression of DHX37 in mouse and human developing testis.** (**a**–**c**) Expression in embryonic mouse gonads at sex-determining stage of 11.5 dpc (17–21 tail somites [ts]). (**a**) Quantitative reverse-transcription polymerase chain reaction (qRT-PCR) analysis of *Dhx37* in wild-type (WT) mouse gonads reveals no significant difference in expression between XX and XY (*p* *=* 0.056, Student’s *t* test). Expression is relative to *Hrpt1*; error bars represent standard error. (**b**) Whole-mount in situ hybridization reveals *Dhx37* expression in WT XY gonad (to the left of the dashed line). Signal appears stronger towards the coelomic surface. (**c**) Immunofluorescence of transverse sections of WT XY gonad (left of the dashed line) showing DHX37 (green), OCT4 (red, primordial germ cells), and DAPI (blue). DHX37 protein is concentrated around the nuclear membrane in most cells, although this is not the case in germ cells (arrows). Cells of the mesonephros (right of the dashed lines) are also positive for *Dhx37*/DHX37 (**b**, **c**). (**d**) DHX37 protein examined in human fetal testis. No protein is observed in gonocytes (marked with arrows), while protein is found in Sertoli cells (marked with asterisk) and in a subpopulation of the interstitial cells. Sample is from a gestational week (GW) 10 fetus. Counterstaining with Mayer’s hematoxylin, scale bar corresponds to 50 µm.

Consistent with a role in testis determination and early testis development, DHX37 is expressed exclusively in somatic cell lineages of the mouse and human gonad during testis determination and development (Fig. 3). The highest concentration of the protein is at the nuclear membrane, although protein is also observed in the cytoplasm and in the nucleolus of some cells (Fig. 3). Analysis of protein expression in different tissues from adults reveals that DHX37 is highly localized at the nuclear membrane in multiple human and mouse cell lines (https://www.proteinatlas.org/ENSG00000150990-DHX37/cell). Although expression was not observed in germ cells in fetal gonads, in adult human testes the protein is mainly localized in spermatogonia (Fig. S3; https://www.proteinatlas.org/ENSG00000150990-DHX37/tissue/testis#img). Single-cell expression analysis of *Nr5a1*^+^ somatic cells of the developing XY mouse gonad also indicates that *Dhx37* is coexpressed with the sex-determining gene *Sox9* in a proportion of cells (Fig. S4). When transfected into HEK 293 cells, the wild-type DHX37 protein and all six mutant proteins showed localization to the nucleolus (Fig. S5).

## DISCUSSION

We provide compelling genetic evidence that specific variants in the DEAH-box protein DHX37, which encodes a putative RNA helicase, are a frequent cause of nonsyndromic 46,XY gonadal dysgenesis as well as TRS. The frequency of pathogenic variants associated with gonadal dysgenesis of 11% is similar to that described previously for each of the genes *SRY*, *NR5A1*, and *MAP3K1*.^10^ Where tested, all pathogenic variants are de novo with one exception, which was inherited from the phenotypically normal mother. This suggests that the penetrance of the variants is high and that the phenotype is sex-limited.

In contrast to the range of phenotypes associated with variants in other testis-determining genes, pathogenic variants involving *DHX37* are associated with TRS but not anorchia. Children with TRS and with either female or ambiguous external genitalia are considered to have had a normal testis determination but have then lost testicular tissue at a critical period during the first 16 weeks of gestation. The genital phenotype may range predominantly from male to female, including marked sex ambiguity depending on the duration of normal testicular function prior to the loss of testicular tissue. Previously, a child with a pathogenic variant in the testis-determining factor NR5A1 (p.V355M) was reported, who presented with bilateral anorchia and reduced penile length; however, his dizygotic twin brother was also heterozygous for the change yet had normal development, suggesting that other genetic factors may be involved.^32^ Here, we found that 25% of children with clinically well-defined TRS carried pathogenic variants in the *DHX37* gene. These data indicate that DHX37 has critical roles not only in early human testis determination but also in the maintenance of testicular tissue during an early phase of testis development. They also establish that 46,XY gonadal dysgenesis and TRS can be part of the same clinical spectrum with the same underlying genetic etiology.

The external genitalia of children carrying *DHX37* pathogenic variants ranged from completely female to male with bilateral cryptorchidism. Four of the 13 children had virilized external genitalia and were raised as male suggesting that functional Leydig cells were present during fetal development. There is no evidence for a genotype/phenotype correlation. For example, five children carried the recurrent p.R308Q pathogenic variant with a phenotype that varied from female with primary amenorrhea to male with micropenis and bilateral cryptorchidism.

The yeast ortholog of DHX37, Dhr1, is required for ribosome biogenesis.^33–35^ Dhr1 is a essential component of the small subunit (SSU) processome, where its activity drives the transition of the SSU processome to pre-40S maturation pathway.^35^ Recently, the human DHX37 protein was also shown to be required for maturation of the small ribosomal subunit in the nucleolus of human HeLa cells by interacting with similar protein partners and small nucleolar RNAs suggesting that this function is conserved in eukaryotes.^36^

Two homozygous variants in *DHX37* have been suggested to cause severe microcephaly, intellectual disability, and cortical atrophy in two unrelated individuals.^37^ However, both variants (p.R487H and p.N419K) are not located within known conserved and functional domains of RecA1 and RecA2. Although there was no statistical evidence in favor of pathogenicity, these pathogenic variants may have resulted in a severe congenital phenotype due to their homozygosity, location within the protein, and/or they may represent hypermorphic or neomorphic pathogenic variants. Further genetic investigations of this syndromic form of congenital microcephaly are required.

Disorders associated with ribosome biogenesis, the ribosomopathies, are an emerging and poorly understood branch of medicine.^38,39^ Indeed, three human ribosomopathies are caused by pathogenic variants in other components of the SSU processome: *UTP4* (MIM 607456) pathogenic variants and North American Indian childhood cirrhosis (NAIC; MIM 604901); *UTP14* (MIM 300508) with infertility, ovarian cancer, and scleroderma; and *EMG1* (MIM 611531) with Bowen–Conradi syndrome (MIM 211180).^38,39^ The mechanism(s) of how a pathogenic variant in a widely expressed protein involved in a basic cellular function can generate a highly specific human phenotype are unknown. However, a zebrafish Dhx37 missense mutant has been reported in association with anomalies in the production of glycine receptor mRNAs, leading to an impact on neuron glycinergic synaptic transmission and altered behavior.^40^ This suggests that DHX37 may be involved in precursor mRNA (pre-mRNA) splicing with substrate specificity for certain transcripts at least in some cell lineages. These data raise the intriguing possibility that some forms of 46,XY DSD, caused by anomalies of early testis development, are also ribosomopathies.

## Supplementary information

Supplementary Information

Supplementary Figure 1
