# Supplementary material for: Pathogenic variants in the DEAH-box RNA helicase DHX37 are a frequent cause of 46,XY gonadal dysgenesis and 46,XY testicular regression syndrome
Source: Genet Med. 2019 Jul 24;22(1):150–9. doi: 10.1038/s41436-019-0606-y (PMC6944638; doi:10.1038/s41436-019-0606-y)
Supplement: Supplementary file 1 — Supplementary Information [file 41436_2019_606_MOESM1_ESM.docx]

| DHX37 EXON 1 | 5’-GCCCCGCCTACTTCTCTAAA-3’  5’-CCACCCGAGAGTCCTGAAG-3’ |
| --- | --- |
| DHX37 EXON 2 | 5’-GCCAAGCCTTGAGAACATCA-3’  5’-CCCTCAGGAGGAGAGAGACC-3’ |
| DHX37 EXON 3 | 5’-CCAGCTTGCTGTGTCTGTTG-3’  5’-TCAGGCCACCATAAAGGTATTT-3’ |
| DHX37 EXON 4 | 5’-TATCTGCAGGAAGGGCATTT-3’  5’-ACATGGAGGCACAGAGAAGG-3’ |
| DHX37 EXON 5 | 5’-TCCCTGTCTCTGTCTCTCCTG-3’  5’-CCCAGGCTCTTATCGACCTT-3’ |
| DHX37 EXON 6 | 5’-CTGCATGGTGAATGTCGAAC-3’  5’-ACAGATGCTGCTCACCTCCT-3’ |
| DHX37 EXON 7 | 5’-TTCCCTTCCCTTCAGAAACC-3’  5’-GGAGTGCTGGATCAACAGGT-3’ |
| DHX37 EXON 8 | 5’-CTCATGTCCATTTCCCACCT-3’  5’-GATTCACAGCCCTGGCACTA-3’ |
| DHX37 EXONS 9/10 | 5’-GGCAGGTCCCACTATTGACT-3’  5’-GGAATAAGGCCAGACAGGTG-3’ |
| DHX37 EXONS 11/12 | 5’-TACTCCTGTCCTCCCCCATT-3’  5’-CTGGACAACGCAGCCTTACT-3’ |
| DHX37 EXON 13 | 5’-CCAATGTTTAGGTGGGGAAA-3’  5’-GTGCCAGGCAGTTTCAAGA-3’ |
| DHX37 EXON 14 | 5’-AGGGCAGCAGAGACAAGAGA-3’  5’-GGAGGGTTCCCTATTCTCCA-3’ |
| DHX37 EXON 15 | 5’-TTGCTACGTCTCCAGCACAC-3’  5’-CCCTTGTTCCTCAGTGGCTA-3’ |
| DHX37 EXON 16 | 5’-GTGAGGAGAATGAGGCAGGA-3’  5’-GGTCACAGGAGCCCAGAAG-3’ |
| DHX37 EXONS 17/18 | 5’-GTTCTTGGCATTCACCCTGT-3’  5’-CCCAGGCTAGAGTGCAGTTG-3’ |
| DHX37 EXON 21 | 5’-CAAGGGAGAAGGAGGGAATG-3’  5’-ACAGCTGAGAGGGAACAAGC-3’ |
| DHX37 EXONS 22/23 | 5’-GAGCACTCTCCCACTTTTGC-3’  5’-CACCCGAGACACACACGTC-3’ |
| DHX37 EXONS 24/25 | 5’-AGTGTCCTGGGCAACCTG-3’  5’-TGTGTGTGGTGCTTTGTCCT-3’ |
| DHX37 EXONS 26/26 | 5’-TTCAGCCCCCTGAGTAACTG-3’  5’-GACCCACTTCCTGAGAGCAG-3’ |

**Supplementary Table 1**. PCR primers used for Sanger sequencing of *DHX37* coding sequences

**Supplementary Table 2.** Variants in the RecA1 or RecA2 domains of the DHX37 protein and the MAF reported in ExAC.

| **Mutation** | **MAF, Population** |
| --- | --- |
| p.Ala311Thr (rs201177773) | 0.000817, South Asian |
| p.Val329Ile (rs112262509) | 0.001442, African |
| p.Thr675Met (rs61757599) | 0.00124, South Asian |
| p.Glu676Asp (rs1206419934) | 0.000008, European (non-Finnish) |
| p.Pro677Ala (rs757085546) | 0.000055, East Asian |
| p.Gly678Ser (rs746007936) | 0.0000353, European (non-Finnish) |
| p.Glu693Lys  (rs374203282) | 0.0001494, European (non-Finnish) |

**Supplementary Result: *In-silico* modelling of rare non-pathogenic DHX37 variants.**

The p.A311T variant is part of motif Ia but is also not directly involved in RNA binding. p.A311T and p.V329I are both buried residues with in the RecA1 while p.G678S is buried within RecA2 domain and these variants are unlikely to have major structural consequences (Fig. S2A). Residues 675 to 677 map to a solvent exposed loop connecting beta sheets and their side chains are pointing away from the ATP binding pocket, which is contrary to the p.R674Q predicted to be causative for gonadal dysgenesis. This suggests that p.T675M, p.E676D and p.P677A retain a catalytically active protein, whilst p.R674Q disrupts function. Finally, residue 693 maps to a solvent exposed section of the RecA2 domain, which is remote from the subsrate binding pockets and the p.E693K mutation is not predicted to alter enzymatic activity . In summary, most of these potentially non-pathogenic variants are solvent exposed rather than having direct interaction with internal components of the DHX37 helicase that may severely disrupt function.

**Supplementary Figure 1. Conservation of DHX37 protein in eukaryotes.** The species names of are indicated and the position of amino acid changes associated with 46,XY gonadal dysgenesis and 46,XY TRS are shown on top. Dashes indicate the sequences were incomplete or gaps introduced in the alignment. Background colors indicate the degree of conservation of the amino acid residues.

**Supplementary Figure 2. *In Silico* modelling of DHX37 RecA1 and RecA2 variants present in the general population and likely non-pathogenic.** Panel (A) shows the position of these variants, highlighted in blue in the RecA1 and RecA2 domains. Panel (B) shows a zoomed in view of p.A311T and p.V329I in ball and stick, highlighted to be buried with a pocket within the RecA1 domain with nearby residues shown as sticks. (C), Residue 674 which interacts with the ATP analog along with its adjacent residues (675-678), with it’s known mutations. In Panel (D), residue 693 is highlighted to interact with a lysine within the RecA2 domain. Dashed lines within figures are shown for selected non-covalent polar interactions.

**Supplementary Figure 3. Expression of DHX37 in human adult testis and cell lines.** Panel A shows DHX37 expression in a human normal 46 yr old male testis, where in contrast to the early developing testis, the DHX37 protein is mainly confined to the spermatogonia of the germ cell lineage (arrows; Bar 100 μm, www.proteinatlas.org).^1^ Panel B shows DHX37 protein (Green) in the breast cancer cell line MCF7 with highest expression on the membrane of the nucleus (DAPI, Blue). In the absence of DAPI staining, Panel C shows areas of discrete nuclear staining (arrows). Microtubules in Panels B and C are labelling in red. Bar 20 μm ([www.proteinatlas.org](http://www.proteinatlas.org)).^15^ Panel D shows the nuclear or perinuclear localisation of the DHX37 protein in the cell lines, RT4, KGN and HEK293.

1. Uhlén, M., Fagerberg, L., Hallström, B.M., Lindskog, C., Oksvold, P., Mardinoglu, A., Sivertsson, Å., Kampf, C., Sjöstedt, E., Asplund, A. et al. (2015). Proteomics. Tissue-based map of the human proteome. Science. *347*, 1260419

**Supplementary Figure 4.** **Single cell expression analysis of Dhx37.** Box plot analysis depicting temporal (E10.5 to E16.5) cell-to-cell variations in gene expression for *Dhx37* and selected sex-determining genes (*Sry*, *Sox9* and *Sox8*) from the single-cell expression analysis of the developing XY mouse gonad.^13^ The maximum, minimum, mean, 99% percentile and 1% percentile points for cell-to-cell expression are presented in the boxplot with the top and bottom of the box representing Q3 and Q1 respectively. Outliers outside of the 1.5-fold IQR are shown. A subgroup of cells expresses *Dhx37* along with the known sex-determining gene *Sox*9 since the box plots representing the distributions of expression for the two genes are similar at every time point analyzed. *Sry* shows transient expression at the time of murine sex determination (E11.5), whereas *Sox8*, which is required for gonadal function in mouse, shows a later expression (E13.5 ono).

**Supplementary Figure 5**. Cellular localisation of mutant and wild-type DHX37 proteins. Human

embryonic kidney (HEK 293-T) cells were transfected with GFP-tagged WT-DHX37 or DHX37 mutant proteins. 48 hours post transfection the cells were fixed and the expression of DHX37 proteins was detected using anti-GFP (green). Slides were also co-stained for the highly conserved nucleolar protein Fibrillarin (red). The nucleus is stained by DAPI (blue). Scale bars are 10 μm. Three mutant proteins are shown as an example but all exhibited the same nucleolar localization.


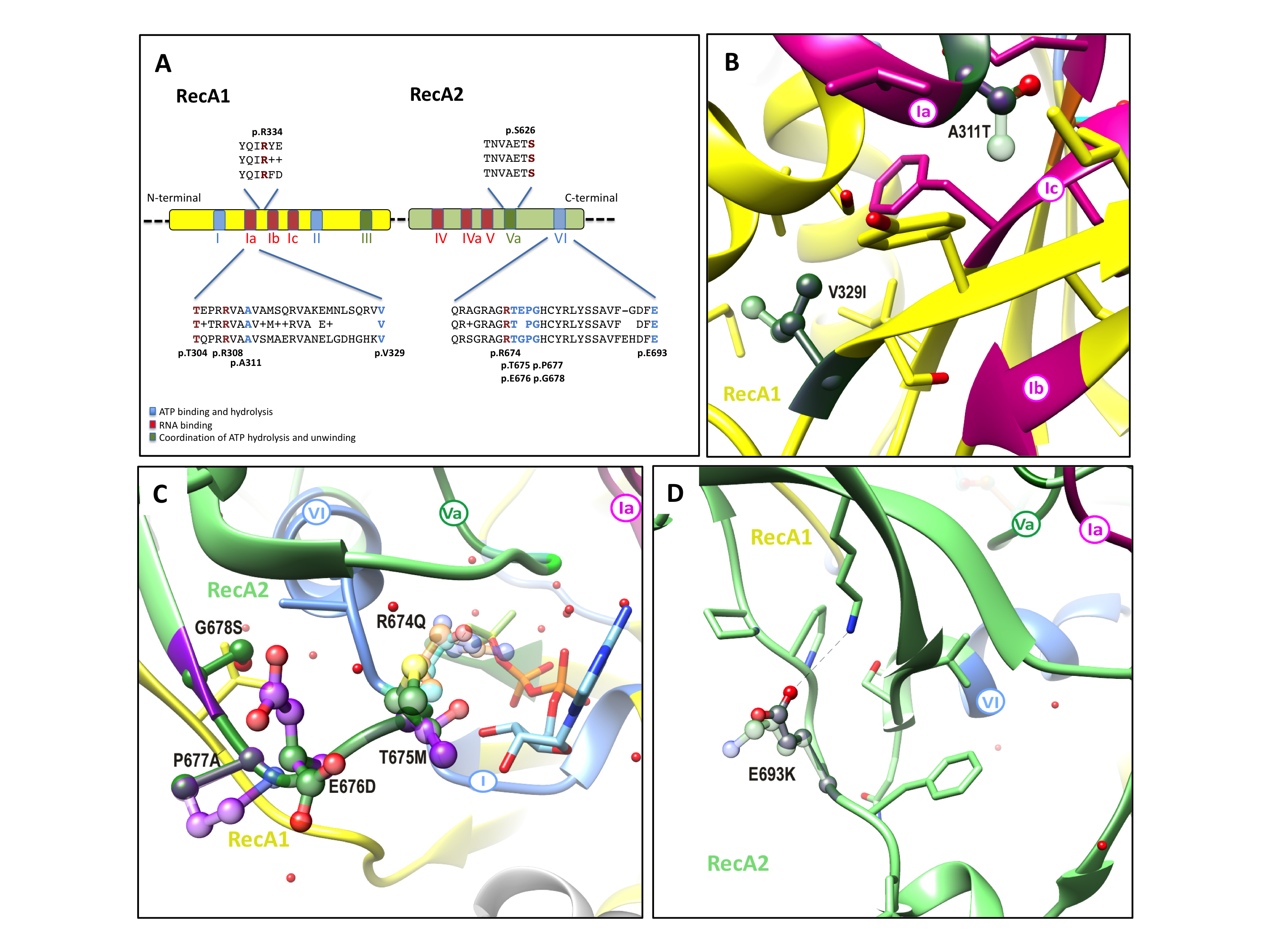


**Supplemental Figure 2.** *In silico* modelling of DHX37 non-pathogenic variants.

**Supplemental Figure 3.** Expression of DHX37 in human adult testis and cell lines.


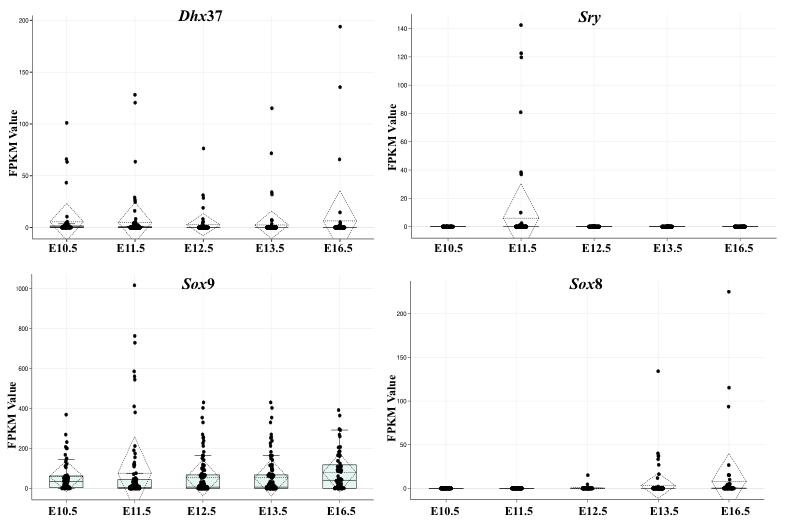


**Supplemental Figure 4.** Box plot analysis depicting temporal (E10.5 to E16.5) cell-to-cell variations in gene expression for *Dhx37* and selected sex-determining genes.

**Supplementary Figure 5. Cellular localisation of mutant and wild-type DHX37 proteins**
