# Supplementary material for: Pathogenic variants in the DEAH-box RNA helicase DHX37 are a frequent cause of 46,XY gonadal dysgenesis and 46,XY testicular regression syndrome
Source: Genet Med. 2019 Jul 24;22(1):150–9. doi: 10.1038/s41436-019-0606-y (PMC6944638; doi:10.1038/s41436-019-0606-y)
Supplement: Supplementary file 2 — Supplementary Figure 1 [file 41436_2019_606_MOESM2_ESM.pdf]

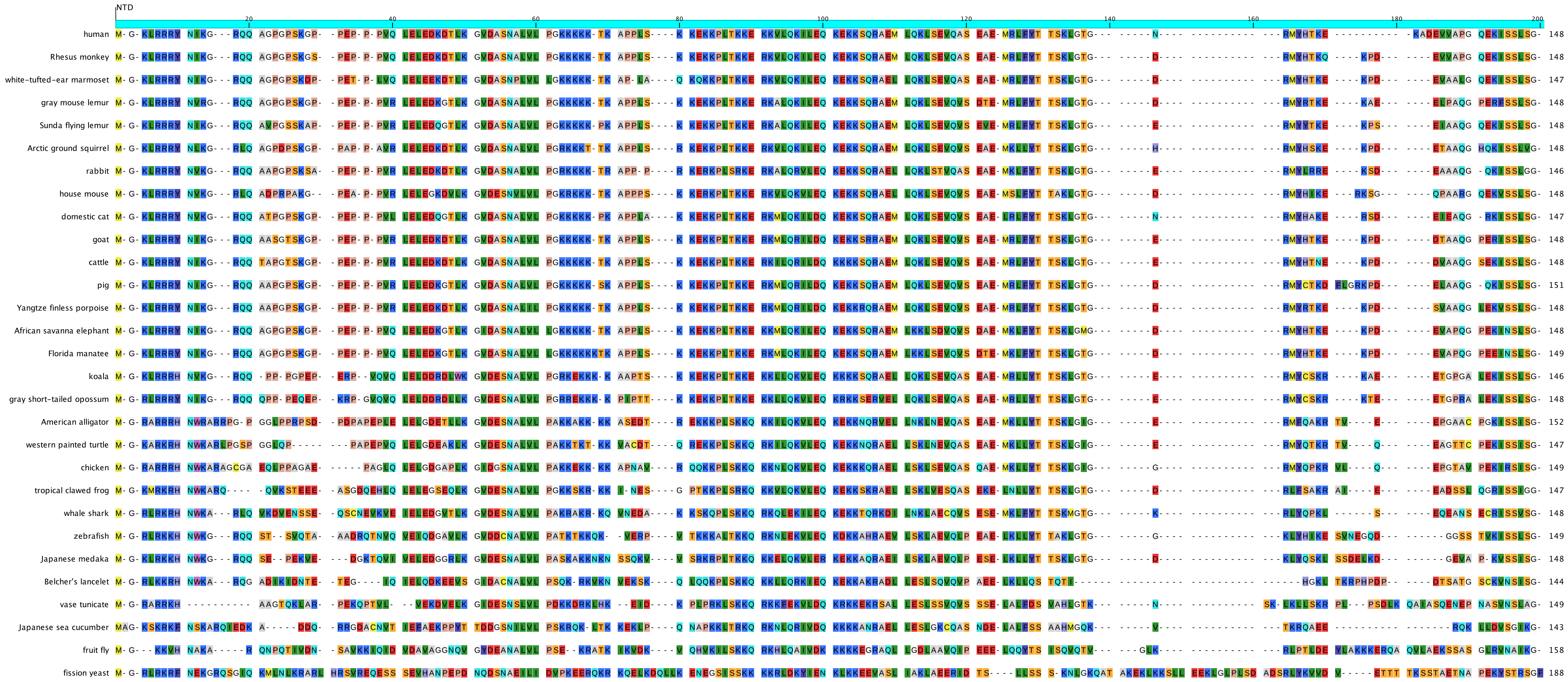
NTD
20
40
60
80
100
120
140
160
180
200
human 148
Rhesus monkey 148
white-tufted-ear marmoset 147
gray mouse lemur 148
Sunda flying lemur 148
Arctic ground squirrel 148
rabbit 146
house mouse 148
domestic cat 147
goat 148
cattle 148
pig 151
Yangtze finless porpoise 148
African savanna elephant 148
Florida manatee 149
koala 146
gray short-tailed opossum 148
American alligator 152
western painted turtle 147
chicken 149
tropical clawed frog 147
whale shark 148
zebrafish 149
Japanese medaka 148
Belcher's lancelet 144
vase tunicate 149
Japanese sea cucumber 143
fruit fly 158
fission yeast 188

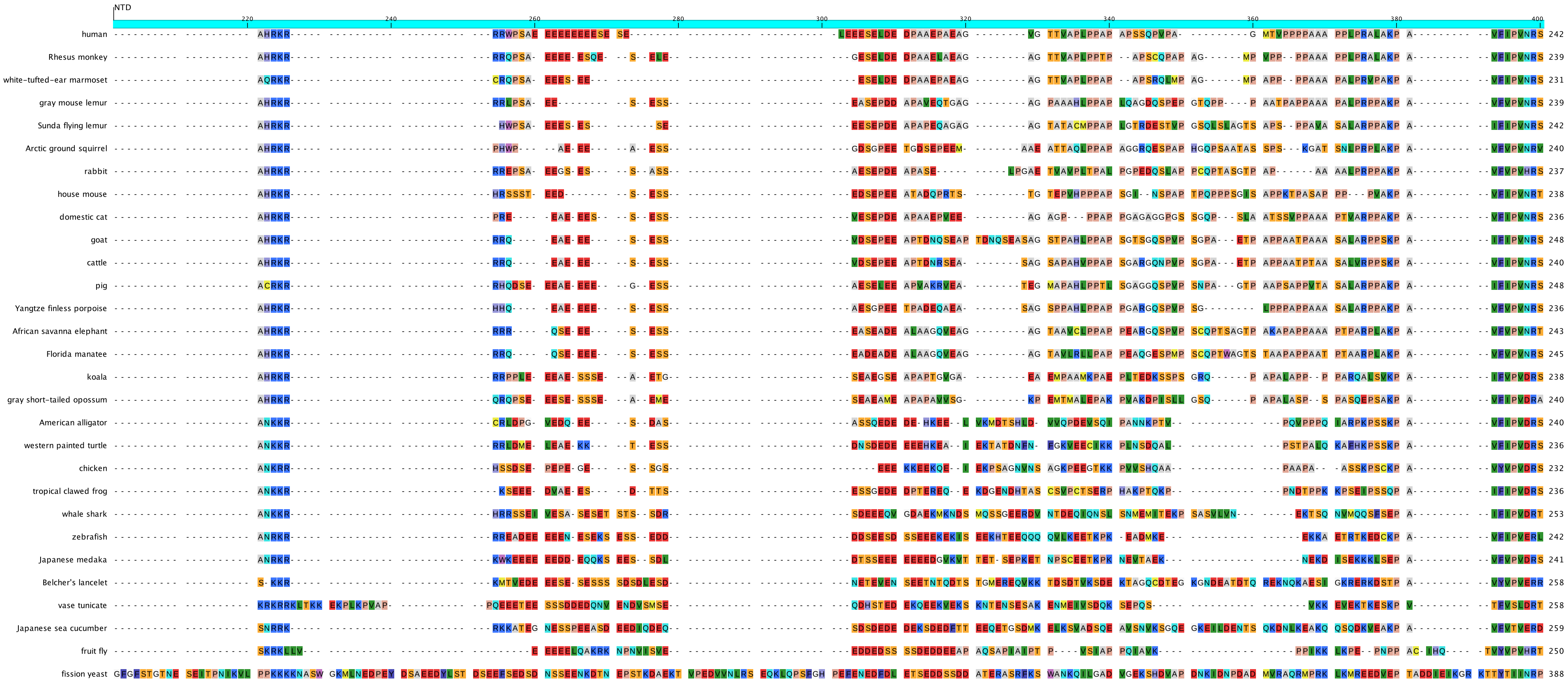
NTD
220
240
260
280
300
320
340
360
380
400
human 242
Rhesus monkey 239
white-tufted-ear marmoset 231
gray mouse lemur 239
Sunda flying lemur 242
Arctic ground squirrel 240
rabbit 237
house mouse 238
domestic cat 236
goat 248
cattle 240
pig 248
Yangtze finless porpoise 236
African savanna elephant 243
Florida manatee 245
koala 238
gray short-tailed opossum 240
American alligator 240
western painted turtle 236
chicken 232
tropical clawed frog 236
whale shark 253
zebrafish 242
Japanese medaka 241
Belcher's lancelet 258
vase tunicate 258
Japanese sea cucumber 259
fruit fly 250
fission yeast 388

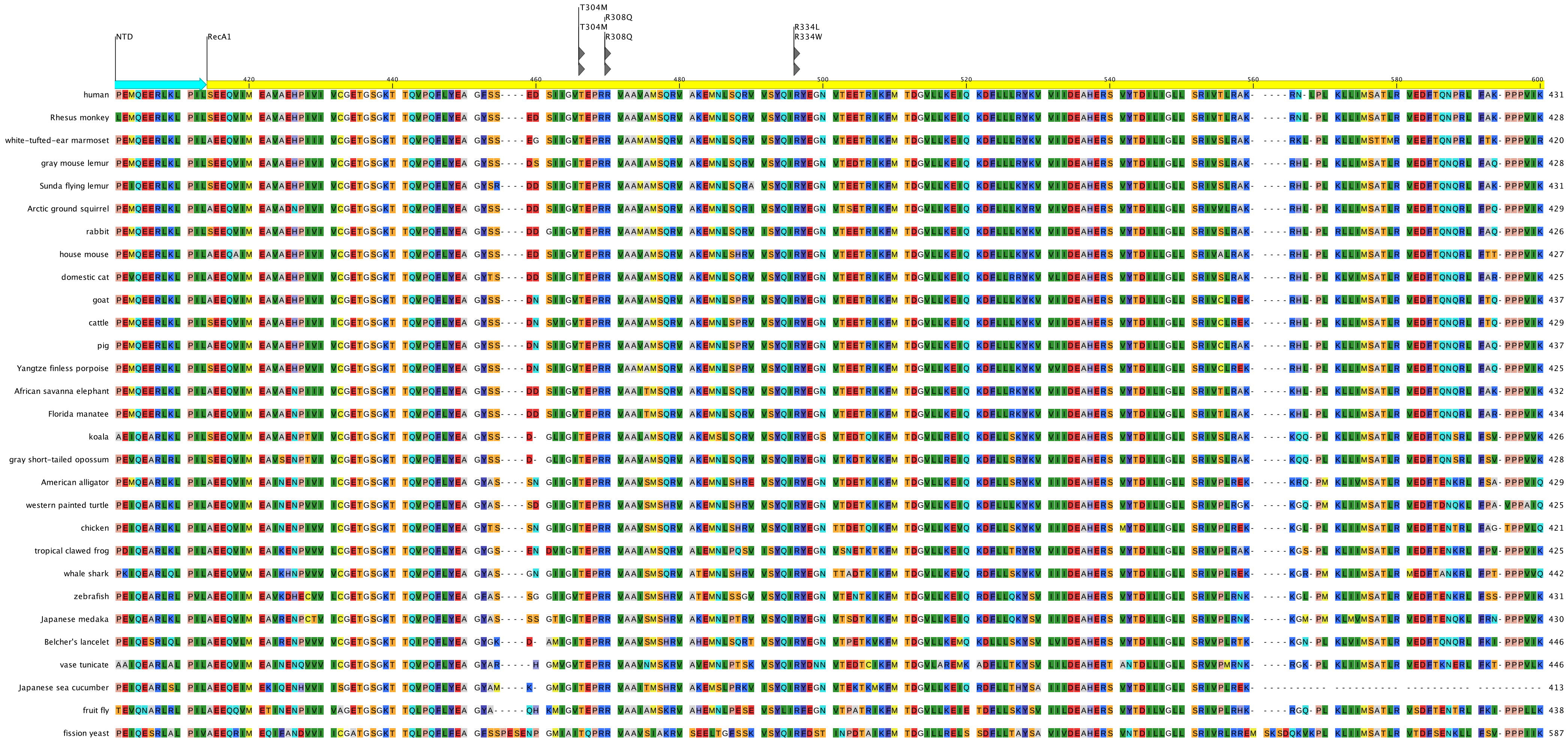
T304M
R308Q
T304M
R308Q
R334L
R334W
NTD
RecA1
420
440
460
480
500
520
540
560
580
600
human
Rhesus monkey
white-tufted-ear marmoset
gray mouse lemur
Sunda flying lemur
Arctic ground squirrel
rabbit
house mouse
domestic cat
goat
cattle
pig
Yangtze finless porpoise
African savanna elephant
Florida manatee
koala
gray short-tailed opossum
American alligator
western painted turtle
chicken
tropical clawed frog
whale shark
zebrafish
Japanese medaka
Belcher's lancelet
vase tunicate
Japanese sea cucumber
fruit fly
fission yeast

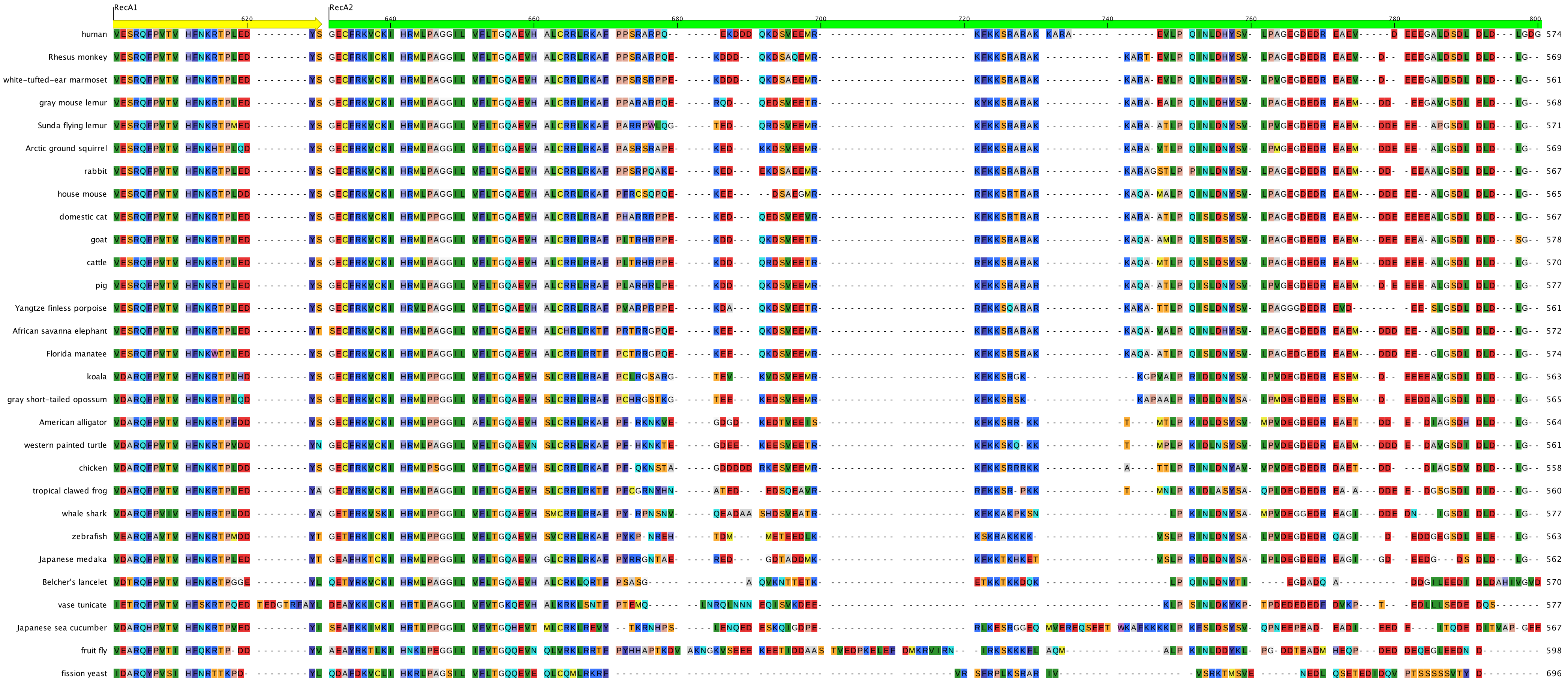
RecA1
RecA2
human 574
Rhesus monkey 569
white-tufted-ear marmoset 561
gray mouse lemur 568
Sunda flying lemur 571
Arctic ground squirrel 569
rabbit 567
house mouse 565
domestic cat 567
goat 578
cattle 570
pig 577
Yangtze finless porpoise 561
African savanna elephant 572
Florida manatee 574
koala 563
gray short-tailed opossum 565
American alligator 564
western painted turtle 561
chicken 558
tropical clawed frog 560
whale shark 577
zebrafish 563
Japanese medaka 562
Belcher's lancelet 570
vase tunicate 577
Japanese sea cucumber 567
fruit fly 598
fission yeast 696

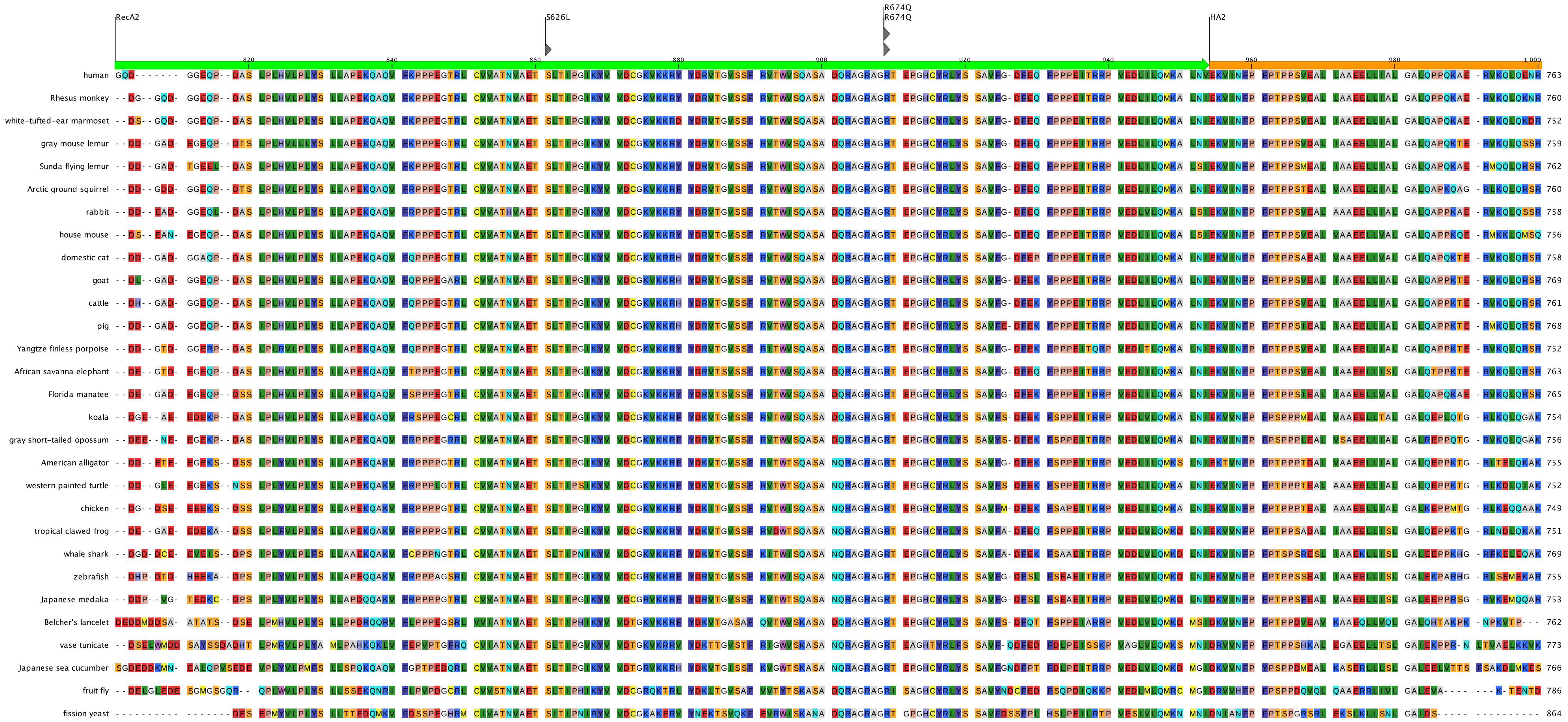
RecA2
S626L
R674Q
R674Q
HA2
human
Rhesus monkey
white-tufted-ear marmoset
gray mouse lemur
Sunda flying lemur
Arctic ground squirrel
rabbit
house mouse
domestic cat
goat
cattle
pig
Yangtze finless porpoise
African savanna elephant
Florida manatee
koala
gray short-tailed opossum
American alligator
western painted turtle
chicken
tropical clawed frog
whale shark
zebrafish
Japanese medaka
Belcher's lancelet
vase tunicate
Japanese sea cucumber
fruit fly
fission yeast

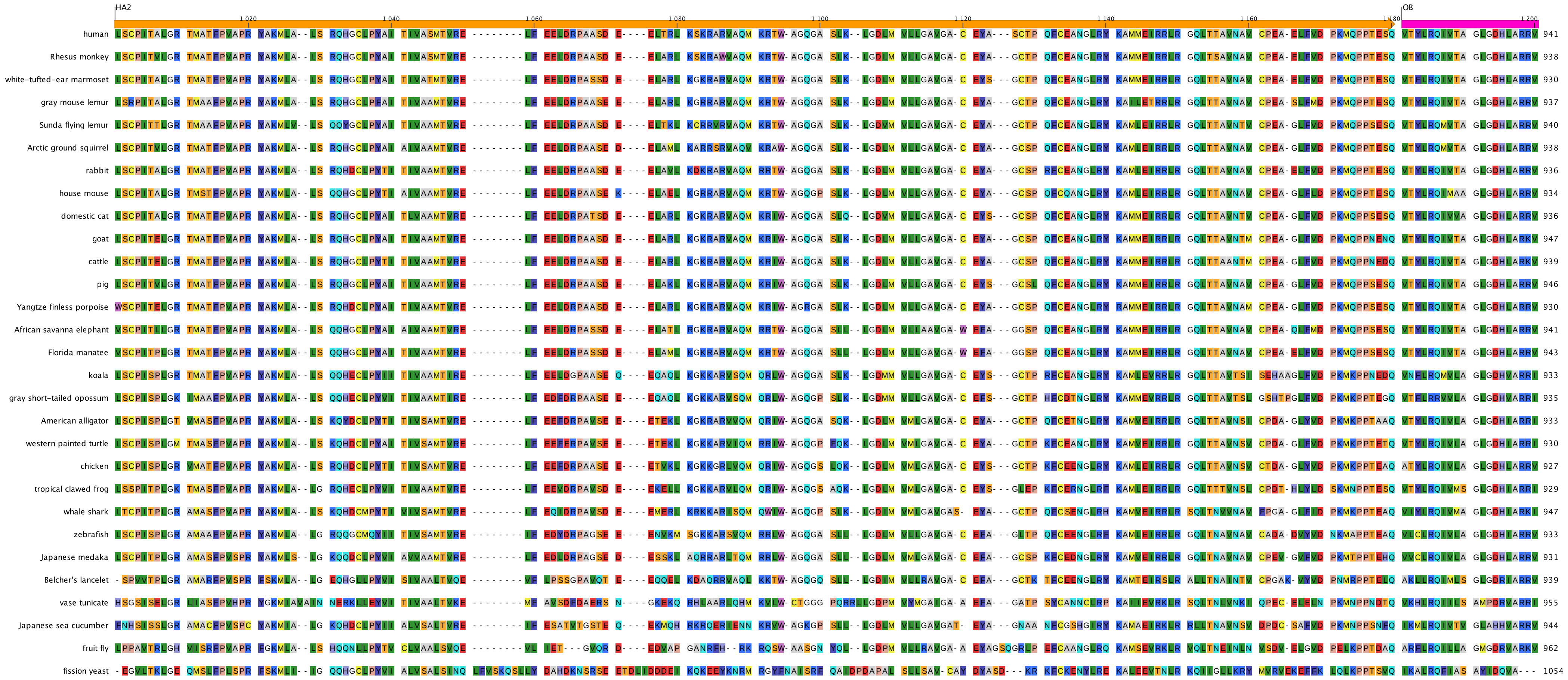
HA2
OB
human
Rhesus monkey
white-tufted-ear marmoset
gray mouse lemur
Sunda flying lemur
Arctic ground squirrel
rabbit
house mouse
domestic cat
goat
cattle
pig
Yangtze finless porpoise
African savanna elephant
Florida manatee
koala
gray short-tailed opossum
American alligator
western painted turtle
chicken
tropical clawed frog
whale shark
zebrafish
Japanese medaka
Belcher's lancelet
vase tunicate
Japanese sea cucumber
fruit fly
fission yeast

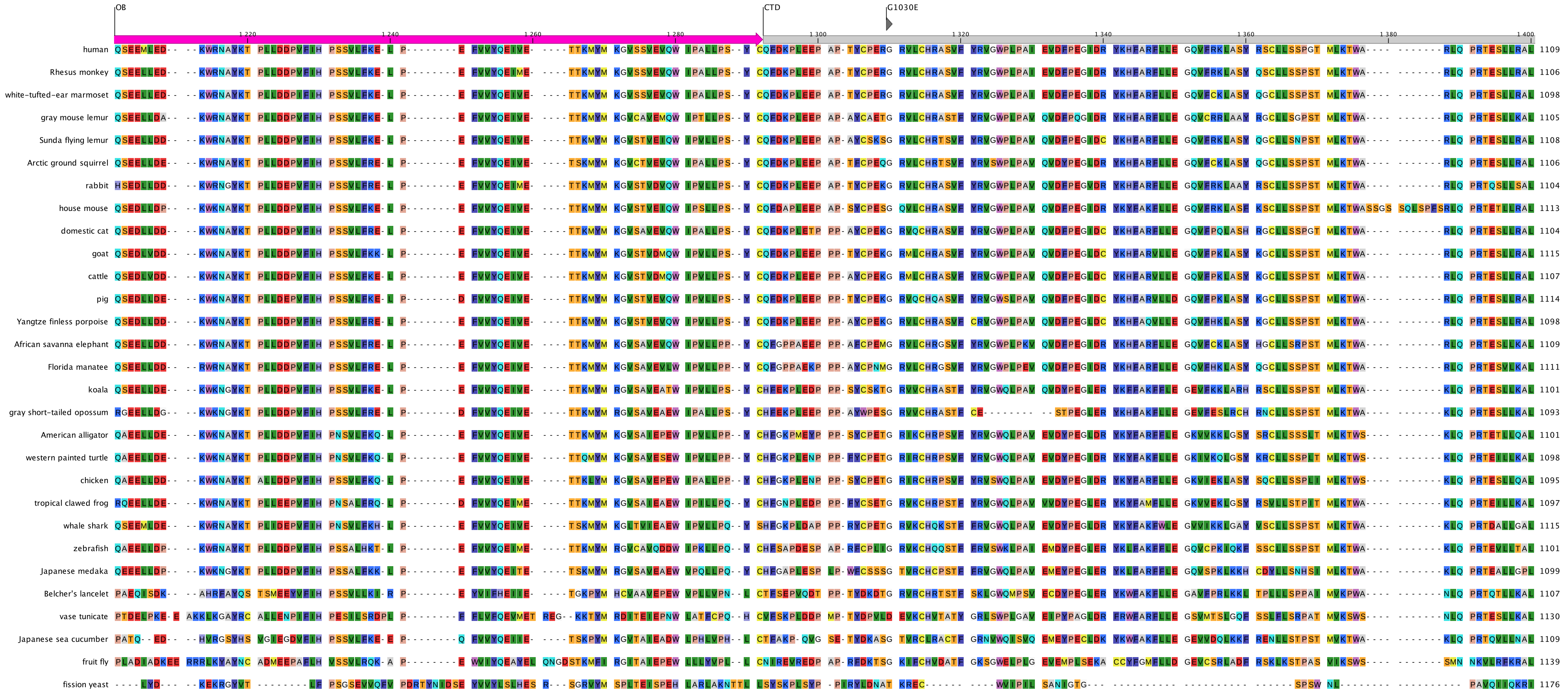
OB
CTD
G1030E
human 1109
Rhesus monkey 1106
white-tufted-ear marmoset 1098
gray mouse lemur 1105
Sunda flying lemur 1108
Arctic ground squirrel 1106
rabbit 1104
house mouse 1113
domestic cat 1104
goat 1115
cattle 1107
pig 1114
Yangtze finless porpoise 1098
African savanna elephant 1109
Florida manatee 1111
koala 1101
gray short-tailed opossum 1093
American alligator 1101
western painted turtle 1098
chicken 1095
tropical clawed frog 1097
whale shark 1115
zebrafish 1101
Japanese medaka 1099
Belcher's lancelet 1107
vase tunicate 1130
Japanese sea cucumber 1109
fruit fly 1139
fission yeast 1176

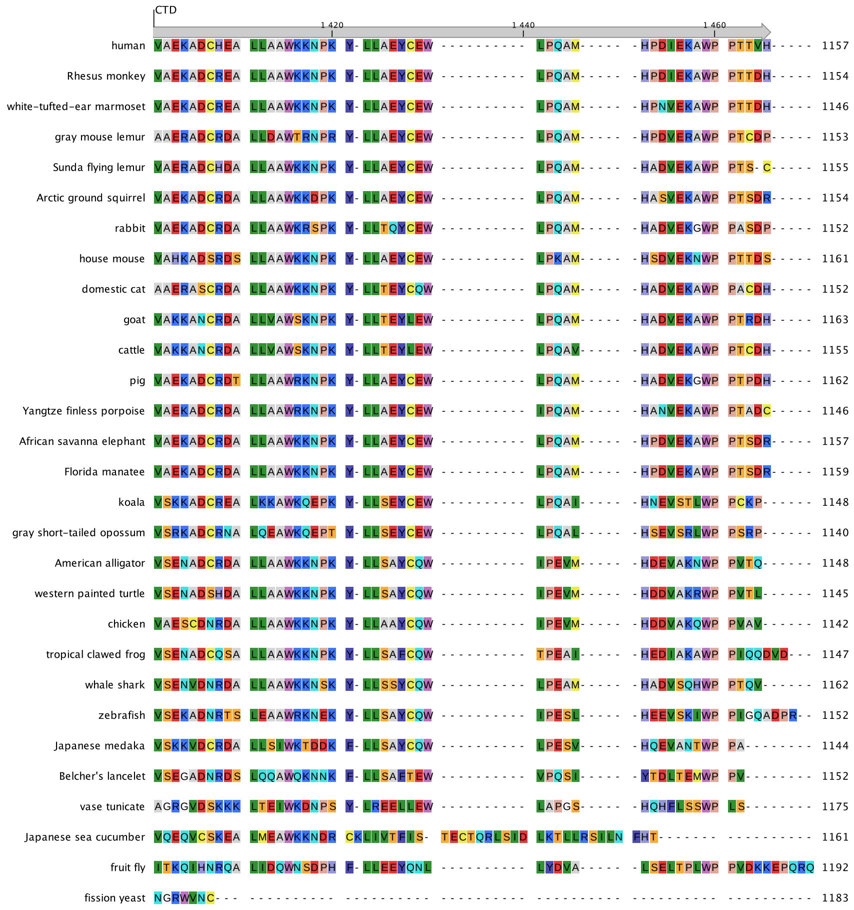
CTD
1 420
1 440
1 460
human 1157
Rhesus monkey 1154
white-tufted-ear marmoset 1146
gray mouse lemur 1153
Sunda flying lemur 1155
Arctic ground squirrel 1154
rabbit 1152
house mouse 1161
domestic cat 1152
goat 1163
cattle 1155
pig 1162
Yangtze finless porpoise 1146
African savanna elephant 1157
Florida manatee 1159
koala 1148
gray short-tailed opossum 1140
American alligator 1148
western painted turtle 1145
chicken 1142
tropical clawed frog 1147
whale shark 1162
zebrafish 1152
Japanese medaka 1144
Belcher's lancelet 1152
vase tunicate 1175
Japanese sea cucumber 1161
fruit fly 1192
fission yeast 1183
